# Relationship between Short- and Mid-Term Glucose Variability and Blood Pressure Profile Parameters: A Scoping Review

**DOI:** 10.3390/jcm12062362

**Published:** 2023-03-18

**Authors:** Elena Vakali, Dimitrios Rigopoulos, Petros C. Dinas, Ioannis-Alexandros Drosatos, Aikaterini G. Theodosiadi, Andriani Vazeou, George Stergiou, Anastasios Kollias

**Affiliations:** 1 Hypertension Center STRIDE-7, National and Kapodistrian University of Athens, School of Medicine, Third Department of Medicine, Sotiria Hospital, 11527 Athens, Greece; 2 Endocrinology–Growth and Development Department, P&A Kyriakou Children’s Hospital, 11527 Athens, Greece; 3 414 Military Hospital for Special Diseases, Penteli, 15236 Athens, Greece; 4 FAME Laboratory, Department of Physical Education and Sports Science, University of Thessaly, 42100 Trikala, Greece; 5 Diabetes Center, A’ Department of Pediatrics, P&A Kyriakou Children’s Hospital, 11527 Athens, Greece

**Keywords:** blood pressure variability, glucose variability, glucose fluctuations, continuous glucose monitoring, ambulatory blood pressure monitoring

## Abstract

*Background.* Increased variability of glucose (GV) and blood pressure (BPV) is linked to a higher risk of macro- and microvascular complications and other hard endpoints. This scoping review aims to summarize the existing evidence regarding the association between the parameters of the blood pressure (BP) profile, especially BPV, with indices of short- and mid-term GV. *Methods*. A literature search was conducted in the MEDLINE/PubMed, Cochrane, Embase, Web of Science, and Wiley Online Library databases. *Results*. The main findings of this review are as follows: (i) 13 studies were included, mainly with small sample sizes; (ii) there was a considerable degree of heterogeneity in the characteristics of the study participants (age range, individuals with normoglycemia, type 1 or 2 diabetes, normal BP, or hypertension), as well as in the methodologies (mainly in terms of the duration of the data collection period) and variability indices examined (mean amplitude of glycemic excursions and coefficient of glucose variation most frequently reported); and (iii) the results were heterogeneous regarding the association between GV and the parameters of the BP profile. *Conclusions*. There is a significant lack of evidence on the association between GV and BPV. Future research implementing a standardized methodology should focus on the determinants, association, and clinical relevance of GV and BPV.

## 1. Background

Some of the components used for cardiovascular risk calculation, including blood pressure (BP) and glucose values, exhibit a continuous dynamic variation in their levels over time. This variation is determined by complex interactions between endogenous physiological circadian rhythms, regulatory neurohormonal and cardiovascular mechanisms, and extrinsic environmental, lifestyle, and behavioral factors [1,2]. Common underlying pathophysiological mechanisms can be hypothesized for similar patterns of variability in these risk factors. In addition, subclinical or established alterations in the structural and functional cardiovascular and renal properties might also contribute to an increased level of variability in these parameters [1,2]. This variability is observed in short-term (within 24 h, minute-to-minute, hour-to-hour, and day-to-night), mid-term (day-to-day over several days), and long-term (visit-to-visit over different months, seasons, and years) periods, representing different mechanisms and interactions [1]. In addition, several parameters of the BP profile, such as specific phenotypes i.e., masked hypertension or a non-dipping BP pattern during nighttime sleep, have been linked to an adverse prognosis [3,4].

The variability of glucose (GV) and BP (BPV) interferes with the accurate assessment of a patient’s glucose and BP status. Moreover, accumulating evidence suggests that both GV and BPV are linked to an increased risk of macro- and microvascular complications and, most importantly, have been associated with adverse hard endpoints [1,5,6,7,8,9]. Despite the acknowledgment of the diagnostic and prognostic implications of the GV and BPV, the use of the latter in clinical practice is still problematic. It was only recently that the time-in-range (TIR) measure was proposed for the assessment of the glycemic control as a continuous glucose-monitoring (CGM)-derived metric, in addition to glycated hemoglobin (HbA1c), which is the gold-standard marker for predicting the relative risk of diabetes complications [10,11]. Furthermore, although the clinical implications of increased BPV are acknowledged, BPV has only research applications at present [12]. Recent studies have linked TIR with both HbA1c and diabetes complications, while CGM or flash-glucose-monitoring (FGM) devices are considered to be the best evaluation tools for this purpose compared to the self-monitoring of blood glucose (SMBG) [13,14]. 

There are few data regarding the association between the parameters of the BP profile (average levels, BPV, phenotypes, and patterns derived from ambulatory monitoring) and the short- and mid-term GV. Apart from a possible common pattern of regulatory mechanisms and behaviors, a combined increased variability in both parameters might be detrimental in terms of micro- and macro-vascular complications. Studies assessing a possible link between GV and BPV are scarce and heterogeneous in terms of the indices used, as well as of population characteristics. The most commonly used indices of GV and BPV, reflecting dispersion, sequence, instability, and specific patterns of the glucose and BP values, are shown in Table 1.

This scoping review aims to summarize the existing evidence regarding the association between indices of short- and mid-term GV and the parameters of the BP profile, especially BPV. 

## 2. Methods

A scoping review was performed according to “The Joanna Briggs Institute Reviewers’ Manual 2015–Methodology for JBI Scoping Reviews” [18]. A preliminary search for relevant scoping reviews was conducted in the following databases: PubMed Central, Embase, Cochrane Library, Web of Science, and Wiley Online Library. No results were retrieved from this search.

### 2.1. Objective and Research Question

The objective of this review was to summarize the current evidence on the association between parameters of the BP profile and the short- and mid-term GV. Specifically, the research question included any type of association (correlation, prevalence of a specific pattern, etc.) between BP profile parameters (average levels, BPV, hypertension phenotypes, and specific patterns, i.e., nocturnal non-dipping) and indices of short- and mid-term GV.

### 2.2. Inclusion Criteria

Eligible studies were considered all those providing details on both BP profile parameters and indices of short- and mid-term GV determined in any kind of population (otherwise healthy individuals, patients with diabetes and/or hypertension and/or other comorbidities, etc.) and including any methodological design. All metrics of GV and BPV presented in Table 1 were considered. Full text, as well as abstract publications were considered eligible. 

### 2.3. Exclusion Criteria

1. Long-term variability was defined by visit-to-visit office-determined glucose, HbA1c, and BP measurements;
2. Use of SMBG for GV assessment or intra-arterial BP recordings for BPV. New technologies offer a wide range of new assessment tools and provide a broader picture of glucose concentrations throughout the day compared to the traditional methods (SMBG). In addition, intra-arterial BP recordings allow for the assessment of very-short-term BPV but their usefulness is mainly restricted to a given research field.

There were no anthropometric or disease-related exclusion criteria.

### 2.4. Search Strategy

The search was conducted in three steps, as proposed by the JBI Manual [18], by two independent reviewers (EV and DR). An initial search was conducted in MEDLINE/PubMed and Cochrane databases in order to identify some related papers and use them as a base to retrieve the appropriate keywords and index terms to search for further studies linking short- or mid-term GV assessed by CGM devices to any parameter of the BP profile. No language, publication date, or study design filters were applied in our search. Additional sources were manually searched for eligible records but no relevant results were retrieved.

In the second step, all identified keywords were used in MEDLINE/PubMed, Cochrane, Embase, Web of Science, and Wiley Online Library, in order to spot all the eligible papers published until 16 January 2023. The search query was modified according to each database’s special search requirements. Details on the searching algorithm can be found in Appendix A. In order to avoid neglecting relevant studies, general terms were preferred with regard to BP. All retrieved papers were processed through Mendeley Citation Manager for further evaluation and an initial check for duplicate articles was conducted. In the process of checking the eligibility of the studies, the Automated Systematic Review software [19] was used in order to support the selection process. All inclusion and exclusion criteria that were implemented are described in the specified sections above. All discrepancies that emerged between the two independent investigators were resolved through consultation with a senior author. The final search strategy for MEDLINE can be found in Appendix B.

After identifying all eligible papers, in the third step of this search, a thorough screening of the reference lists of the included papers for additional eligible articles was conducted. A more detailed presentation of the search algorithm can be found in Appendix A.

During the search, most citations were excluded based on their titles and abstracts, but 324 records were manually screened due to the lack of details in the databases in which these were found. From these citations, none were found to be eligible according to the inclusion criteria. Records for which an abstract was not available were designated for a full-text review in order to verify their eligibility. For articles whose full text could not be obtained through our institutional resources, the original plan was to contact the corresponding authors for further details; however, no such article was detected through our search.

## 3. Extraction of the Results

The extraction of the results was performed according to the following chart:

- Identify the main author and publication year;
- Report the aims of the study;
- Define the study population with respect to the following parameters:
  ▪ Number of participants;
  ▪ Participants’ main characteristics (age, sex, and body mass index);
  ▪ Percentage of participants with cardiovascular risk factors (diabetes, hypertension, dyslipidemia, smoking);
  ▪ Percentage of participants treated for cardiovascular risk factors;
  ▪ Level of glycemic and BP control;
  ▪ Duration of diabetes and hypertension.
- Determine the type of CGM device used:
  ▪ CGM;
  ▪ FGM.
- Determine the BP assessment method (office, ambulatory, or home monitoring);
- Determine the GV and BPV indices that were calculated;
- Ascertain the key findings related to the research question.

## 4. Presentation of the Results

Summary characteristics of the included studies are presented in Table 2.

Most of the included studies incorporated a relatively small number of participants (<70) and only two of them included a large sample size (*n* > 200). Interestingly, almost half of the included studies were abstract publications with limited published data and no follow-up publication. All studies were published in the last 15 years, which is reasonable due to the recent advances in CGM systems. It should be mentioned that only one study used flash-glucose-monitoring technology, while the rest of them used the CGM system. The studies included individuals with both type 1 and type 2 diabetes and individuals with a normal glucose status. Regarding BP status, most studies included individuals with normal BP levels.

Table 3 presents the main characteristics and findings of the included studies. 

Not all studies provided details regarding the methodologies used and the populations studied. Of the 13 included studies, 7 studies [21,23,25,27,29,31,32] (54%) reported the same CGM methodology for GV assessment despite heterogeneity in the monitoring period (ranging from 48 h to 7 days). One study used the DexCom G4 Platinum technology [28] whereas seven studies used Medtronic glucose sensors [21,23,25,27,29,31,32]. One study used an intermittently scanned CGM device (using flash technology—FGM) for 14 days [30]. Regarding the BP assessment methodology, seven studies used ambulatory BP monitoring (ABPM) for the assessment of the participants’ BP profiles [20,24,25,27,28,31,32]. Five studies used office BP measurements [21,22,26,29,30], most of them with a significant lack of details, and a single study reported the use of self-measurements [23]. 

**Figure 1:**
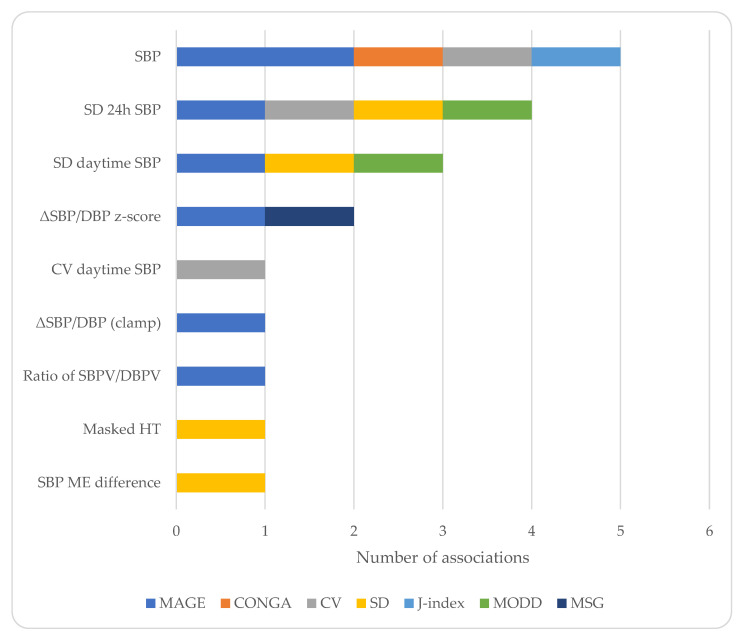
Charted representation of the extracted data. Only significant associations are shown. DBP: diastolic blood pressure, HT: hypertension, ME: morning–evening, MSG: mean sensor glucose, SBP: systolic blood pressure, SBPV/DBPV: systolic/diastolic blood pressure variability, SD: standard deviation, and ΔSBP/DBP: change in SBP/DBP.

There was also a high degree of heterogeneity regarding the indices reported (Figure 1) and the type of associations between the GV and BP parameters examined (Table 3). The most commonly studied index of GV was MAGE [20,21,22,23,25,27,32], as well as the SD of glucose [23,30,31,32], while for BP parameters, average levels [20,22,23,24,26,28], SD [32], and dipping status were studied [27]. A few studies reported positive associations between GV indices and average BP levels [22,24,26]. Two studies reported associations between the indices of GV and BPV [24,32], and one study reported that an increased GV determined the presence of masked hypertension [31]. Other studies did not report associations [23,27] and one study reported an inverse correlation between MAGE and the indices of BPV [25]. 

Regarding the association between GV or BPV and the indices of asymptomatic organ-damage, one study reported no association between MAGE and pulse wave velocity [21], whereas another one related that MAGE was a determinant of microalbuminuria in patients with type 2 diabetes [22].

## 5. Discussion

This review intended to summarize all the available evidence connecting short- and mid-term GV with the parameters of the BP profile such as average levels, BPV, dipping status, hypertension phenotypes. The main findings are as follows: (i) the available data regarding the association between GV and BPV are scarce; (ii) there is a considerable degree of heterogeneity in the characteristics of the study populations, in the methodology—mainly the duration of the data collection period—and the variability indices examined, and in the analyses performed; and (iii) only two studies reported significant correlations between similar indices of GV and BPV [24,32]. 

This field remains quite understudied considering the recent advances in the understanding of the pathophysiology and importance of variability and the technological developments allowing for its assessment. Thus, the included studies were limited, and several of them were published in the form of abstract presentations. In addition, the lack of standardization of the methodology for the assessment of GV and BPV resulted in a large degree of heterogeneity in the indices examined and the methodology employed for their determination. It is interesting to note that, rarely, the same indices of variability were examined within the same study for assessing GV and BPV. In fact, two studies assessed the correlation between the same indices of GV and BPV, one in otherwise healthy individuals [32] and the other in individuals with diabetes and hypertension [24], with both reporting positive associations. The first study could imply common regulatory neurohormonal mechanisms under healthy conditions [32]. Moreover, the second study could suggest that pathological conditions, such as diabetes and hypertension, are accompanied by similar patterns of disruptions in these regulatory mechanisms, as reflected in the positive association between GV and BPV. The other studies included several populations, mainly with type 1 or 2 diabetes, and reported either significant associations between BP indices—mainly average BP levels—and GV [20,21,22,24,25,26,30,31] or a lack of such associations [23,27]. Another important limitation of the included studies was the variation in the monitoring period for assessing GV, which ranged from a few hours to several days. According to a recent international consensus statement, a confident interpretation of CGM metrics requires 14 consecutive days of CGM data with at least 70% of the data collected during that time period, which is predictive of the 3-month glycemic status [11]. Interestingly, most of the included studies used CGM for up to 72 h.

The methodology employed in the assessment of GV and BPV is of paramount importance. Ideally, continuous monitoring and sampling would be the reference method for evaluating dynamic measures. Unfortunately, all the available assessment methods allow for intermittent sampling during the monitoring period. In CGM, the sensor measures glucose every 5–10 s but averages these values every 5 min over a 2- to 6-day period for a single sensor [33]. On the other hand, ABPM allows for BP measurements every 15 to 30 min, usually for a 24 h period [1]. Invasive BP measurements allow for beat-to-beat BP recordings, but these are not feasible in clinical practice. Thus, CGM and ABPM are the only available and validated methods that allow for the assessment of short-term variability in a clinical context. Home and office BP measurements allow for the assessment of mid- and long-term BPV, respectively [1].

Variation in glucose and BP levels is determined by endogenous circadian rhythms and regulatory neurohormonal mechanisms in response to extrinsic environmental and behavioral factors. In several disease settings (type 1 or 2 diabetes, hypertension, etc.), the underlying pathological mechanisms might contribute to further variation, yet the pattern of the derangement of the regulatory mechanisms, as well as the drug treatment characteristics, might account for significant differences in the variability patterns. For example, in type 1 diabetes, the metabolic control network is completely degraded due to lack of endogenous insulin secretion and a need for external insulin replacement, whereas in type 2 diabetes, the metabolic network structure is largely preserved [34]. Indeed, GV is significantly higher in type 1 vs. type 2 diabetes, mainly due to the higher frequency of hypoglycemic episodes [35,36]. On the other hand, current treatment options for type 2 diabetes are largely devoid of the side effect of hypoglycemia, which means that GV in these patients is largely driven by hyperglycemic excursions [36].

The potential relationship between GV and BPV could be better understood by considering the pathophysiological mechanisms that might determine such fluctuations. Both GV and BPV seem to be influenced to a great degree by the autonomic nervous system and oxidative stress [32]. More specifically, there have been reports of an inverse association between baroreceptor reflex sensitivity and GV in patients with type 2 diabetes [37] and between reduced baroreceptor reflex and BPV [37]. Additionally, a vicious circle can be supported in which atherosclerosis is linked to oxidative stress induced by increased GV, which, in turn, can contribute to increased BPV and further deteriorate the existing oxidative stress levels [38]. In addition, GV enhances the production of advanced glycation end-products, which play a fundamental role in endothelial damage [39], while BPV seems to be actively related to reduced endothelial function, even though the exact mechanism that connects BPV and endothelial damage has not yet been clarified [40]. The potential connection between GV and BPV stresses the importance of underlying mechanisms but also of underlying subclinical target organ damage that can increase GV and BPV in a similar manner.

Apart from the association between GV and BPV, their coexistence could aggravate cardiovascular and renal damage. Thus, identifying individuals with increased GV and BPV would allow for more effective risk stratification. Furthermore, people with type 1 diabetes could benefit the most from future research in this field, especially if we consider that most technological devices available in diabetes practice (which allow for the assessment of GV) are designed for and used by people diagnosed with type 1 diabetes.

## 6. Conclusions and Perspectives

This review identified a limited and heterogeneous amount of evidence regarding the association between BP profile parameters and short- and mid-term GV. The preliminary reports indicate associations between GV and BPV both in healthy conditions and in disease settings. As already mentioned, the advances in the technological devices employed in glucose monitoring, as well as BP monitoring can be of assistance in future research. Nowadays, those devices are being used widely, and our understanding of their functions and applications grows by the day. Particularly, regarding CGM systems, the automatically extracted reports generate several GV indices without the need for further calculations. Examples include TIR and CV, which can be easily accessible, can be potentially useful in clinical practice, and may show great potential in research. Analyses including such indices could be directly applied to clinical practice without the need for extra calculations, thereby saving time and resources. The widespread use of such technology accompanied by automated and standardized reports including indices of interest could lead to a better understanding of several mechanisms with great clinical potential. Wearable devices allowing non-invasive glucose monitoring or cuffless devices performing frequent BP measurements could provide detailed assessments of glucose and BP profiles including GV and BPV [41,42,43]; however, such devices have not been validated with respect to their accuracy according to established protocols and, most importantly, in terms of their clinical utility and intended use [41]. The association between GV and BPV could highlight the importance of several common underlying mechanisms. Furthermore, the assessment of GV and BPV could lead to risk reclassification and the identification of high-risk patients, thereby facilitating the development and implementation of prompt therapeutic strategies in the context of primary prevention. Physicians who treat diabetic and hypertensive patients should consider a more detailed diagnostic assessment in cases of increased GV and/or BPV, as well as a meticulous total cardiovascular risk assessment in the presence of a combination of increased GV and BPV.

## Figures and Tables

**Table 1:** Commonly used indices of glycemic and blood pressure short- and mid-term variability [1,12,15,16,17].

Index	Definition
**Indices of both glucose and blood pressure variability**	
Standard Deviation (SD)	Dispersion of the raw values (square root of variance)
Coefficient of variation (CV)	Extent of dispersion in relation to mean value (SD/mean value) × 100
Average real variability (ARV)	Average of absolute differences between consecutive values
**Indices of blood pressure variability**	
Nighttime dipping	Percentage of decrease in nighttime blood pressure
**Indices of glucose variability**	
Mean amplitude of glycemic excursions (MAGE)	Mean differences from peaks to nadirs
Continuous overlapping net glycemic action (CONGA)	Difference between a current blood glucose reading and a reading taken hours earlier
Mean of daily differences (MODD)	Absolute differences between two glucose values measured at the same time with a 24 h interval
Time in range (TIR)	Percentage of time per day within target glucose range (70–180 mg/dL)

**Table 2:** Summary characteristics of the included studies.

Characteristics	Number (*n* = 13)	Percentage (%)
**Publication year**		
2008	3	23
2011–2013	2	15
2016–2018	3	23
2020–2021	5	39
**Publication type**		
Journal article	7	54
Abstract publication	6	46
**Population size**		
<30	3	23
31–70	8	60
>200	2	15
**GV assessment methodology**		
CGM	12	92
FGM	1	8
**BP assessment methodology**		
Ambulatory BP monitoring	7	54
Office BP measurements	5	38
Self-monitoring	1	8
Diabetes status *		
T1D	6	46
T2D	5	39
NGT	4	30
IGT/IFG	2	15
Hypertension status *		
HTN	2	15
Masked HTN	1	8
Normal BP	10	67

* The percentages are overlapping because some studies used multiple populations. CGM: Continuous Glucose Monitoring, FGM: Flash Glucose Monitoring, T1D: Type 1 Diabetes, T2D: Type 2 Diabetes, NGT: Normal Glucose Tolerance, IGT: Impaired Glucose Tolerance, IFG: Impaired Fasting Glucose, and HTN: Hypertension.

**Table 3:** Data extraction table.

Study	Population	Age ± SD (Years)	Males (%)	Methodology for GV	BP Assessment	Main Findings
Golicka 2008 [20]	*n* = 44 T1D normotensives	Range: 11–18	NR	CGM	24 h ABPM	MAGE associated with nocturnal SBP
Gordin 2008 [21]	*n* = 22 T1D	25.9 ± 5.6	100	72 h CGM	Office BP and aortic BP (applanation tonometry) during a 2 h hyperglycemic clamp	MAGE associated with aortic BP difference between acute hyperglycemia and baseline values at 0′
Zhou 2008 [22]	*n* = 176 T2D *n* = 48 NGT	NR	NR	CGM	Office BP	MAGE correlated with SBP
Borg 2011 [23]	*n* = 268 T1D *n*= 159 T2D	48.7 ± 13.2	48	48 h CGM performed at baseline and at 4-week intervals	Self-monitoring of BP	Glucose variability indices (SD, CONGA, MAGE) not associated with SBP/DBP
Sakamoto 2013 [24]	*n* = 64 DM hypertensives	53 ± 12	NR	48 h CGM	48 h ABPM	CV of glucose associated with 24 h SBP and CV of awake SBP
Rosales 2016 [25]	*n* = 11 NGT	Range: 30–40	NR	72 h CGM	24 h ABPM	MAGE inversely associated with indices of BP variability
Rezki 2017 [26]	*n* = 19 IGT normotensives *n* = 15 T2D normotensives	NR	NR	CGM 3 h after breakfast	Office aortic BP (applanation tonometry) 1 h after breakfast	CONGA and J-index associated with peripheral and aortic SBP
Jaiswal 2018 [27]	*n* = 41 T1D normotensives	34 ± 13	39	5-day CGM	24 h ABPM	CV of glucose and MAGE not associated with BP dipping
De Backer 2018 [28]	*n* = 68 T1D	NR	NR	7-day CGM	24 h ABPM	Mean but not SD of glucose associated with nocturnal DBP
Karnebeek 2020 [29]	*n* = 33 overweight (32 NGT, 1 IGT)	12.5 ± 3.2	39	48 h CGM	BP measured every 3 min for 1.5 h	Lifestyle-induced changes in CONGA and CV not correlated with changes in SBP/DBP z-scores
Shimizu 2021 [30]	*n* = 40 hospitalized with CVD (47.5% DM, 67.5% hypertension)	70.0 ± 11.0	78	Up to 14 days FGM	BP measured twice daily	SD of nighttime glucose correlated with morning minus evening SBP in DM patients
Homhuan 2021 [31]	*n* = 28 T1D normal office BP (27% masked HT)	13.8 ± 3.8	33	7-day CGM	24 h ABPM	Higher SD of glucose in masked HT vs. normotensionCV of glucose > 36% predicted masked HT
Sezer 2021 [32]	*n* = 27 NGT normotensives	23.8 ± 2.7	33	48 h CGM	24 h ABPM	SD of 24 h ambulatory SBP correlated with MAGE, MODD, SD of glucose. SD of daytime ambulatory SBP correlated with MAGE and MODD.

ABPM: Ambulatory Blood Pressure Monitoring, BP: Blood Pressure, BPV: Blood Pressure Variability, CGM: Continuous Glucose Monitoring, CONGA: Continuous Overlapping Net Glycemic Action, CV: Coefficient of Variation, CVD: Cardiovascular Disease, DBP: Diastolic Blood Pressure, DM: Diabetes Mellitus, FGM: Flash Glucose Monitoring, GV: Glucose Variability, h: hour, HT: Hypertension, IGT: Impaired Glucose Tolerance, MAGE: Mean Amplitude of Daily Excursions, MBG: Mean daily Blood Glucose, ME: Morning–Evening, MODD: Mean Of Daily Differences in Glucose, NGT: Normal Glucose Tolerance (euglycemia), SBP: Systolic Blood Pressure, SD: Standard Deviation, T1D: Type 1 Diabetes, and T2D: Type 2 Diabetes.

**Figure A1:**
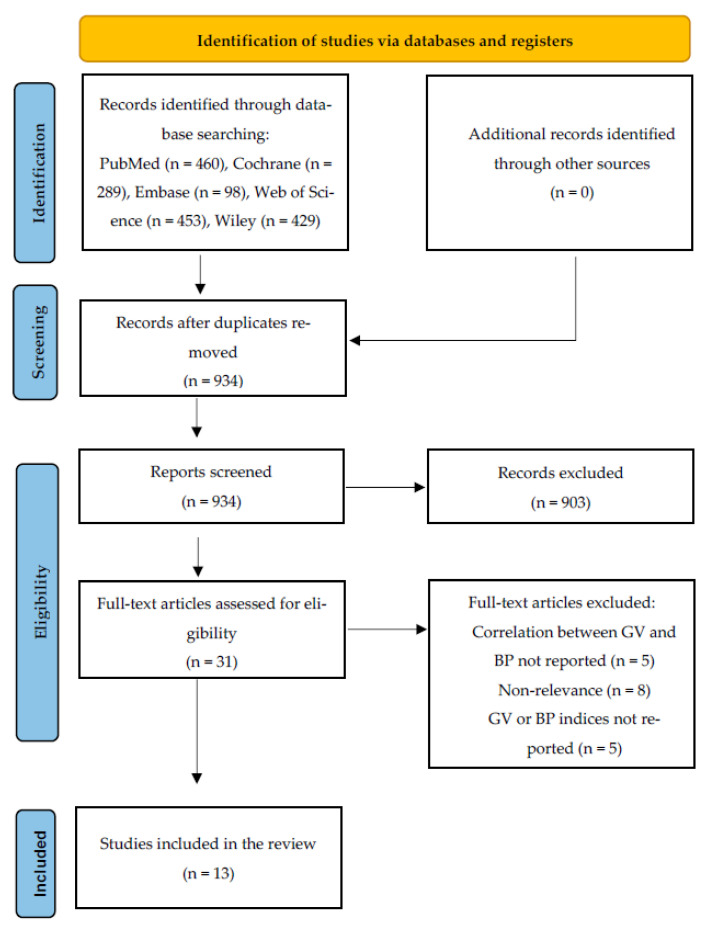
PRISMA Flow Diagram for the Scoping Review Process.

## Data Availability

Not applicable.

## Appendix B

Final Search Strategy for MEDLINE

- (((“blood pressure”[Title/Abstract]) OR (bp[Title/Abstract])) OR (“Arterial Pressure”[Mesh])) OR (hypertension[Title/Abstract])
- ((“glucose alteration*”[Title/Abstract]) OR (“glycemic alteration*”[Title/Abstract])) OR (“glycaemic alteration*”[Title/Abstract])
- ((((“glucose fluctuation*”[Title/Abstract]) OR (“glycemic fluctuation*”[Title/Abstract])) OR (“glycaemic fluctuation*”[Title/Abstract])) OR (“blood sugar fluctuation*”[Title/Abstract])) OR (“blood-sugar fluctuation*”[Title/Abstract])
- (((((“glucose variability”[Title/Abstract]) OR (‘“gv”[Title/Abstract])) OR (“glycemic variability”[Title/Abstract])) OR (“glycaemic variability”[Title/Abstract])) OR (“blood sugar variability”[Title/Abstract])) OR (“blood-sugar variability”[Title/Abstract])
- (((cgm[Title/Abstract]) OR (“continuous glucose monitor*”[Title/Abstract])) OR (fgm[Title/Abstract])) OR (“flash glucose monitor*”[Title/Abstract])
- (((((((cgm[Title/Abstract]) OR (“continuous glucose monitoring”[Title/Abstract])) OR (fgm[Title/Abstract])) OR (“flash glucose monitoring”[Title/Abstract])) OR ((((((“glucose variability”[Title/Abstract]) OR (‘“gv”[Title/Abstract])) OR (“glycemic variability”[Title/Abstract])) OR (“glycaemic variability”[Title/Abstract])) OR (“blood sugar variability”[Title/Abstract])) OR (“blood-sugar variability”[Title/Abstract]))) OR (((((“glucose fluctuation*”[Title/Abstract]) OR (“glycemic fluctuation*”[Title/Abstract])) OR (“glycaemic fluctuation*”[Title/Abstract])) OR (“blood sugar fluctuation*”[Title/Abstract])) OR (“blood-sugar fluctuation*”[Title/Abstract]))) OR (((“glucose alteration*”[Title/Abstract]) OR (“glycemic alteration*”[Title/Abstract])) OR (“glycaemic alteration*”[Title/Abstract]))) AND ((((“blood pressure”[Title/Abstract]) OR (bp[Title/Abstract])) OR (“Arterial Pressure”[Mesh])) OR (hypertension[Title/Abstract]))
